# Dementia and communication comorbidities within the context of caregiver support systems: a scoping review

**DOI:** 10.1186/s12877-025-06622-0

**Published:** 2025-12-04

**Authors:** Nisha Dhanda, Emil Basil Scaria

**Affiliations:** 1 Department of Applied Health Sciences, College of Medicine and Health, University of Birmingham, Birmingham, UK; 2 Department of Applied Health Sciences, College of Medicine and Health, University of Birmingham, Birmingham, Dubai United Arab Emirates

**Keywords:** Communication, Dementia, Hearing loss, Caregiver support systems, Communication comorbidities, Quality of life

## Abstract

**Background:**

Dementia is a growing global health concern affecting millions of people worldwide. Communication difficulties are a hallmark feature that pose challenges for both people with living dementia (PLWD) and caregivers. These challenges encompass spoken and unspoken communication and can lead to social isolation and diminished quality of life. Caregivers also face significant burdens owing to communication difficulties, stress, and burnout.

**Aims:**

This review aimed to explore the existing literature on dementia and communication comorbidities within the context of caregiver support systems. By synthesising current evidence, this review aimed to identify gaps in knowledge, highlight effective interventions, and inform future research and clinical practice aimed at improving the communication experiences of individuals with dementia and supporting their caregivers.

**Methods:**

The review methodology was guided by the PRISMA-ScR framework. A search strategy using relevant keywords was developed, and searches were conducted using PubMed and EMBASE without any limitations. Two independent reviewers screened the titles and abstracts and full texts using predetermined eligibility criteria. Data extraction was conducted by two reviewers and disagreements were resolved through discussion. The extracted data were grouped based on common themes and narratively synthesised.

**Results:**

A total of 228 articles were screened at the title and abstract levels, and 120 full-text articles were assessed for eligibility. 23 eligible studies were included in the narrative synthesis. Most of these studies were conducted in the United States of America (47.8%) and Europe (43.5%). The studies used various designs, with an almost equal number of cohort studies (30%), qualitative studies (26%), and experimental studies (26%). The key themes identified were as follows: adult day service use reducing caregiver burden, educational interventions reducing caregiver burden, supportive technologies, caregiver counselling, perspectives of informal caregivers and PLWD, perceptions of staff caregivers. Only one study addressed hearing loss as a communication comorbidity.

**Conclusion:**

The findings highlight the potential value of adult day-care services, caregiver counselling, educational interventions, and supportive technologies in dementia care. Future research that focuses on specific interventions tailored to dementia and communication comorbidity needs and integrating them into care plans can reduce the caregiver burden.

## Introduction

Dementia represents a significant global public health challenge, with an estimated 50 million individuals affected worldwide, a number projected to triple by 2050 [1]. Among the myriad of symptoms associated with dementia, communication difficulties are particularly burdensome for individuals with dementia and their caregivers. Effective communication is essential for maintaining relationships, accessing care, and preserving a sense of dignity and autonomy in individuals living with dementia [2]. However, as the condition progresses, language deficits, impaired comprehension, and changes in social behaviour often impede effective communication [3], which can vary according to the type of dementia.

Communication challenges in dementia are multifaceted, which encompass both expressive and receptive aspects of language as well as hearing impairment and non-verbal forms of communication [4]. It is estimated that aphasia is associated with 25% of all frontotemporal dementia [5]. Hearing loss is considered the largest modifiable risk factor for dementia, and accounts for 8% of cases [6]. Most PLWD also demonstrate anomia and other language deficits [7]. These communication impairments not only impact the individual's ability to convey their needs and preferences, but also contribute to frustration, social isolation, and diminished quality of life [8]. Unmanaged hearing impairment in people living with dementia can lead to or maintain feelings of social isolation, particularly in residential care settings [9].

In the context of dementia care, the burden of communication difficulties extends to family caregivers, who often play a central role in supporting individuals with dementia [10]. Caregivers face numerous challenges, including managing behavioural symptoms, coordinating medical care, and providing emotional support, all of which are exacerbated by communication breakdowns [11]. Moreover, the stress and burden associated with caregiving can negatively affect the caregiver's own physical and mental health, leading to increased rates of depression, anxiety, and caregiver burnout [12].

Recognising the intertwined nature of dementia, communication, and caregiving, efforts to support individuals living with dementia must extend beyond medical interventions to encompass holistic, person-centred approaches that address communication needs and enhance caregiver resilience [13]. Caregiver support programmes that provide education, training, respite care, and psychosocial interventions have shown promise in improving caregiver well-being and enhancing the quality of care provided to individuals with dementia [14].

Considering the growing prevalence of dementia and the complex interplay between communication impairments and caregiver support needs, this scoping review aimed to explore the existing literature on dementia and communication comorbidities within the context of caregiver support systems. By synthesising current evidence, this review seeks to identify gaps in knowledge, highlight effective interventions, and inform future research and clinical practice aimed at improving the communication experiences of individuals with dementia and supporting their caregivers.

The objectives of this review are as follows:

1. Summarise existing literature on strategies employed to support caregivers in the context of dementia and communication comorbidities.
2. Assess the effectiveness of interventions aimed at improving communication between caregivers and individuals with dementia and communication comorbidities.
3. Provide recommendations for future research and the development of tailored interventions to better support caregivers in this context.

## Methods

This scoping review was conducted following PRISMA Extension for Scoping Reviews (PRISMA-ScR) [15]. This review aimed to summarise existing evidence and provide recommendations for how caregivers of people living with dementia and associated comorbidities can be adequately supported. The review was conducted following the 5-stage framework proposed by Levac et.al [16].

### Stage 1: identifying the research question

The objective of this study was to review the existing literature on strategies to support caregivers of PLWD and communication comorbidities, and to examine the effectiveness of such strategies and challenges with their implementation.

The following research question was formulated to address the objectives of this study.

What strategies are employed to support caregivers in managing individuals living with dementia and communication comorbidities?

### Stage 2: identifying databases and relevant studies.

PubMed and Embase databases were searched using tailored search strategies from their inception to 28 February 2024. These two databases were chosen for their comprehensive coverage. Pubmed encompasses MEDLINE and contains millions of studies covering a broad range of biomedical and health sciences research. Embase was searched to further increase the coverage and capture more relevant research on dementia and caregiving. The search strategies used Medical Subject Headings terms for PubMed and Emtree terms for Embase, as well as free words related to topics such as dementia, communication comorbidities, and caregiver support systems. These terms were combined appropriately using Boolean operators such as AND and OR. No limits were applied for the year of publication to capture the most recent evidence during the searches. However, the searches were limited to English language articles only. The search strategy for each database, including Emtree terms for Embase and MeSH headings for Pubmed have been provided in Appendix A. The reference lists of previously published reviews and the included studies were manually searched to identify relevant studies.

### Stage 3: study selection

The following inclusion and exclusion criteria were used to select studies for this review:

#### Inclusion criteria

- Studies focusing on older adults living with dementia and their communication challenges.
- Research exploring communication comorbidities, such as aphasia and unmanaged hearing loss in older adults living with dementia.
- Studies examining caregiver support systems or interventions for older adults living with dementia.
- Research investigating the impact of caregiver support on communication outcomes in individuals with dementia.
- Publications in peer-reviewed journals.
- Studies published in English.
- Research articles, reviews, meta-analyses, case studies, and other scholarly work.

#### Exclusion criteria

- Studies not related to dementia or communication disorders.
- Research not addressing caregiver support systems or interventions.
- Publications not available in English.
- Non-peer-reviewed sources, such as conference abstracts, posters, or dissertations.
- Studies focusing solely on the non-communicative aspects of dementia (e.g. behavioural symptoms).
- Publications with insufficient data or irrelevant review scope

The search results were imported into Covidence, a web-based review-management platform [17]. Duplicates were then identified and removed. Two independent reviewers (ND and ES) performed title and abstract screening using inclusion criteria to identify eligible studies. Disagreements were resolved by discussion. No new studies were included from the reference lists of included studies. All studies deemed eligible and those that could not be excluded were subjected to full-text screening performed by two independent reviewers (ND and ES). Disagreements were resolved by discussion.

### Stage 4: data extraction

A data extraction form was created on Covidence and piloted in two studies. The extraction form included items such as study title, author(s), publication year, geographical origin of the study, aims/purposes, study design and methodology, interventions (if any), details, outcomes and details, and key findings or conclusions relevant to the research question of the review. Data extraction was performed by two independent researchers (ND and ES). Disagreements were resolved by discussion. Once completed, the data were exported to Microsoft Excel [18].

### Stage 5: collating, summarising, and reporting the results

The extracted data was narratively synthesised by the researchers. The narrative synthesis adhered to the framework outlined by Popay et al. [19], which involves four iterative stages—developing a preliminary synthesis, exploring relationships across studies, assessing the robustness of the synthesis, and producing a structured narrative report. The researchers analysed the included studies to identify common themes to help explain the literature landscape of how caregivers of PLWD and associated comorbidities can be adequately supported.

## Results

**Fig. 1:**
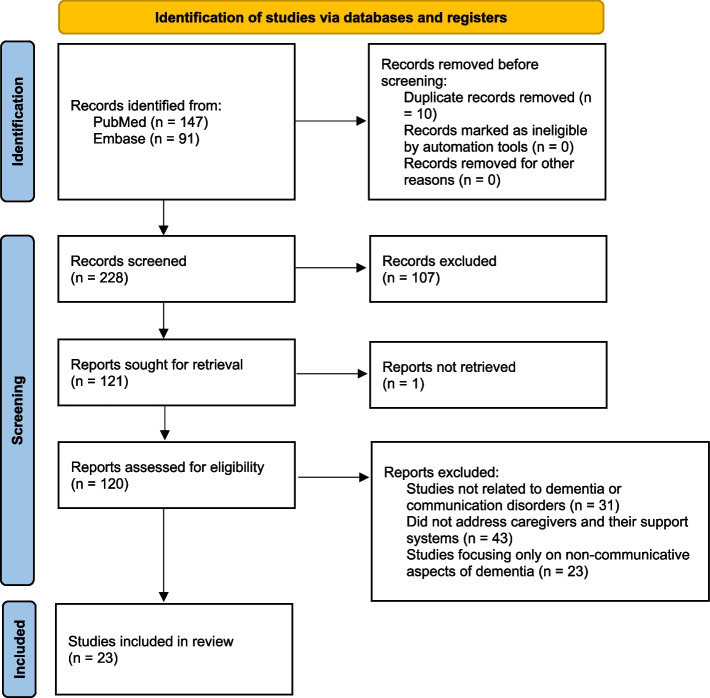
PRISMA flowchart showing study selection process

The study screening and selection process is summarised using a PRISMA flowchart (Fig. 1).

There were 23 studies included in the final review. The studies were geographically varied, with 11 conducted in North America, 10 in Europe, one in New Zealand, and one in Japan. There were six experimental studies, two cross-sectional studies, seven cohort studies, six qualitative studies, one mixed methods study, and one integrative review Table 1.

**Table 1:** Included studies with study designs and geographical locations grouped by themes

Themes	Author (Year)	Study design	Geographical location
ADS use reduces caregiver burden, stress and depression	Tretteteig et al. [20]	Integrative review	Norway
	Honjo et al. [21]	Cohort	Japan
	Parker et al. [22]	Cohort	USA
	Ivey et al. [23]	Cohort	USA
	Mamo et al. [24]	Qualitative	USA
	Liu et al. [25]	Cohort	Switzerland
	Leggett et al. [26]	Cohort	USA
	Orellana et al. [27]	Mixed methods	United Kingdom
	Boafo et al. [28]	Qualitative	USA
	Rokstad et al. [29]	Quasi-experimental	Norway
	Logsdon et al. [30]	Cohort	USA
	Maseda et al. [31]	Cross-sectional	Spain
Educational interventions that reduced caregiver burden	Tamayo-Morales et al. [32]	Randomized controlled trial	Spain
	Bonner et al. [33]	Quasi-experimental	USA
	Tewary et al. [34]	Quasi-experimental	USA
Supportive technologies	Marx et al. [35]	Qualitative	USA
	De Cola et al. [36]	Cohort	Italy
	Liang et al. [37]	Pilot Randomized controlled trial	New Zealand
Caregiver counselling	Behrndt et al. [38]	Randomized controlled trial	Germany
Perspectives of informal caregivers and PLWD	Von Kutzleben et al. [39]	Cross-sectional	Germany
	Faeo et al. [40]	Qualitative	Norway
Perceptions of staff caregivers	Dabelko-Schoeny et al. [41]	Qualitative	USA
Caregiver respite through activities for PLWD	Holden et al. [42]	Qualitative	USA

Key themes identified from the studies were as follows: adult day services (ADS) use reduced caregiver burden, stress, and depression; educational interventions that reduced caregiver burden; supportive technologies; caregiver counselling; perspectives of informal caregivers and PLWD; perceptions of staff caregivers; caregiver respite through activities for PLWD.

### ADS use reduces caregiver burden, stress and depression

Tretteteig et al. [20] conducted an integrative literature review and found day care centres (DCC) that offer respite and support services can provide family caregivers with a sense of safety and relief, lower carer load, and boost motivation for their position as carers. Respite experience is determined by the quality of care provided at the DCC and how well the service satisfies the family caregivers’ needs for flexibility, support, information, and responsibility sharing. This becomes more prominent when a PLWD has additional communication challenges such as hearing loss. Of the 19 studies reviewed by Tretteteig et al. [20], they identified a gap between a family caregiver’s expectations of a DCC and the actual ability of the DCC to reduce their mental load, burden, and feelings of anxiety for the family caregiver. Therefore, they concluded the need for more qualitative research to explore the expectations and needs of family caregivers of PLWD in detail.

The indirect benefits of day care services for family caregivers were outlined by another study [22], which found that adult day programmes provide respite, allowing carers to address self-care requirements while minimising the likelihood of carers missing a doctor's appointment. These findings indicate that adult day services may promote favourable health behaviours in caregivers and should be included as part of comprehensive dementia care for families. However, Black carers missed more appointments than their White counterparts, regardless of the frequency of adult day service use. The demonstrated inequity must be further explored.

Three of the included studies [28–30] demonstrated that the expertise and resources within adult day centres helped to reduce the burden on caregivers, increased respite and reassurance among caregivers, and reduced distress. Maseda et al. [31] also found that there was a significant improvement in the mental well-being of caregivers when they received support.

Adult day services not only provide respite but also incorporate structured communication supports, such as care-planning meetings and staff-mediated updates, which enhance caregivers’ understanding of communication needs [20, 28, 43]. These interactions help caregivers adapt strategies for engaging PLWD, reducing frustration and perceived burden. Additionally, social and recreational activities within ADS, such as group conversation circles and music-based programs, were reported to maintain communication abilities, indirectly supporting caregiver well-being [27, 30, 44]. These findings suggest that the benefits of ADS extend beyond respite to include improved communication dynamics, which is a critical factor in reducing caregiver stress. But it is important to consider the acoustic environment during the social and recreational activities, to ensure that unmanaged hearing impairment is not a hindrance or obstacle to activities designed to enhance cognitive stimulation [9].

### Physical health benefits to carers

A cohort study [25] aimed to investigate the relationship between daily stress biomarkers and functional health over time in dementia family caregivers. They also examined how care transitions and the use of adult-day services affect the long-term relationship between baseline stress biomarkers and functional health. Initially, carers submitted five saliva samples every day over an 8-day diary trial, with each carer having a different number of adult day service days per week. Two longitudinal follow-ups were conducted at 6 and 12 months to assess adult day service use, care transitions, and carer functional health. Care transitions and the total number of adult day service days per week at baseline were viewed as mediators of the relationship between stress biomarkers and health over time. The data revealed that while none of the stress biomarkers had substantial impacts on functional health, trajectories of functional limitations over time were related to daily stress biomarkers in the setting of care transitions. Previous research investigating the relationship between biomarkers and health change used a considerably larger sample size, an index score based on numerous biomarkers, and performance-based measures of physical health, such as balance and gait. Possible explanations for the null results on the main effects of stress biomarkers on functional health include a limited sample size, a single type of stress biomarker, and self-reported functional status.

Another study [23] demonstrated the importance of providing respite to family caregivers, which lowers exposure to care-related stressors, and stress management skills that can assist caregivers in managing daily pain. However, it is difficult to ascertain what a clinically meaningful decline in pain would mean for each person, and the nature of self-reporting makes internal validity challenging.

### Mental health benefits to carers

From the qualitative interviews conducted by Orellana et al. [27], family caregivers reported benefiting from attending day centres. Day centres emerged as life-enriching gateways to companionship, activities, the outside world, practical support, information, other services, the community, and enjoyment for people who had experienced loss, were socially isolated and unable to go out without support.

Moreover, Holden et al. [42] found that social engagement activities by community-based service providers for PLWDprovided respite for caregivers. Behrndt et al. [38] found that when family caregivers were provided with adequate counselling on stress reduction and self-management techniques, it helped caregivers cope with challenging behaviours and reduced caregiver burden.

### Benefits to PLWD

Honjo et al. [21] investigated the role of adult day services within Japan's public nursing care system, particularly in relation to its impact on individuals with Alzheimer's disease. The specific influence of adult day services on the progression of Alzheimer's disease remains uncertain. The researchers conducted a retrospective analysis involving 161 patients diagnosed with Alzheimer's disease, utilising available Mini-Mental State Examination (MMSE) scores. The patients were categorised into two groups: those who engaged with adult day services (*n* = 106) and those who did not (*n* = 55). Subsequently, the researchers compared the MMSE scores of these groups from the initial memory clinic visit to a follow-up at the 6-month mark. Although there were no significant differences observed in terms of gender and the number of family members between the two groups, it was noted that the non-day service group tended to be younger, more educated, and initially scored higher on the MMSE. However, at the 6-month follow-up, day service users exhibited a notable improvement in MMSE scores, indicating enhanced cognitive function. Interestingly, the frequency of day service use did not appear to significantly influence MMSE scores. Nonetheless, after approximately 6 months, day service utilisation showed a significant positive impact on the cognitive function of patients with Alzheimer’s Disease. Whilst the findings of this study indicate the potential for a non-pharmacological therapeutic approach for Alzheimer's patients, there are limitations of the MMSE as a sensitive enough measure for global cognition. Additionally, the Japanese sample population may exhibit different prognoses, experiences, and characteristics of the disease compared to Western populations because of fundamental differences in diet, lifestyle and values.

### Hearing loss as a communication comorbidity

Only one study [24] included in the review explicitly considered hearing loss or additional communication comorbidities for PLWD. This qualitative study was conducted to assess the hearing loss burden and communication needs of participants and staff at a day health centre to create effective staff training and communication interventions that addressed the group's observed and reported needs. The researchers investigated the functional effects and perceived burdens of hearing loss among participants at the day health centre. By observing interactions, they identified various situations that can inform the development of practical training programmes to enhance communication within the day health centre environment. Additionally, insights gleaned from staff focus groups shed light on which training topics would be most engaging for them. For instance, one suggested training topic emerged from discussions on how to differentiate between hearing difficulties and cognitive impairments during communication breakdowns. They posed the use of headset amplifiers during the focus groups as a viable option for aiding communication. Staff members involved in the intake/enrolment process suggested that headset amplifiers should be available for one-on-one and small group consultations, particularly for individuals and families considering regular day care attendance. The consensus among staff was that if personal care aides at the day health recognised the usefulness of amplifiers, they would be more inclined to utilise them.

The authors concluded that a significant portion of older adults attending day services likely experience hearing loss. The staff expressed a desire to learn how to identify hearing loss through communication and behavioural cues in older adults, and they welcomed the prospect of training in communication techniques and amplification tools to enhance communication. To fully maximise social engagement opportunities at the day health centre, addressing and managing hearing loss should be an integral aspect of personalised care plans. The findings offer insights into better equipping staff to communicate effectively with participants who have hearing impairments.

### Educational interventions to reduce caregiver burden

Three studies used RCTs to investigate educational interventions that reduced caregiver burden.

In one study [32], caregivers were trained in the Antecedent-Behavior-Consequence (ABC) model of functional behaviour analysis. The intervention group received eight weekly group sessions of 90 min duration at adult day care centres or health centres. They were educated about behavioural consequences, antecedents to disruptive behaviours, communication skills and management of disruptive behaviours. Evaluations were performed at baseline and 6 months after randomisation in both control and intervention groups. The results suggested decrease in the frequency of disruptive behaviours and showed a marked reduction in caregiver burden and depression.

A study [33] conducted in the USA used advance care treatment plan (ACT-Plan), a group based education intervention for caregivers that provided knowledge on cardiopulmonary resuscitation (CPR), mechanical ventilation (MV) and tube feeding (TF). African American caregivers of dementia participated in a 4-week ACT-Plan or attention control condition intervention. Assessments were made pre-test and post-test, revealing that caregivers attending the ACT-Plan intervention had increased knowledge of dementia, CPR, MV and TF, and improved self-efficacy compared to those in the other group.

The third study [34] assessed a modified group-delivery sleep education program (NITE-AD) that educated caregivers on sleep hygiene, exercise and managing light exposure found that it reduced caregiver burden and depression. However, this study only recruited 7 participants from 2 adult day care centres in the USA.

### Supportive technologies

The supportive technologies identified by three studies included a web-based application to support caregivers, a telehealth service, and a companion robot. In one study [35], WeCareAdvisor, a web-based application was assessed for its ability to assist caregivers in managing and tracking behavioural and psychological symptoms of dementia. Focus groups and semi-structured interviews were conducted with family caregivers. It concluded that caregivers would prefer a user-friendly tool with all requisite information available in one place, helping them deal with behavioural and psychological symptoms of dementia.

Another study [36] looked at telemonitoring of vital parameters and tele-counselling of PLWD, which was found to improve the behaviours of elderly PLWD, increase their spare time, and significantly reduce caregiver stress burden.

A companion robot, Paro, was assessed as an option to reduce caregiver burden in a study [37] from New Zealand. Over the course of 6 weeks, they found that the robot was able to enhance affective and social outcomes in PLWD, and thereby reduce caregiver burden. However, it has to be noted that the PLWD that were most responsive to Paro had relatively better cognitive functioning.

### Perspectives of informal caregivers and dementia patients

Two studies explored the perspectives of informal caregivers or PLWD and their attitudes towards home-care services, adult day centres and assistive technologies. One study [39] used a cross-sectional survey assess perceptions of informal caregivers to care arrangements. It found that care arrangements were often a mix of informal and formal services but informal caregivers assumed greater responsibility. Nursing services, day care and respite care were the most commonly used formal services. The other study [40] explored the perceptions of PLWDs towards volunteer support, assistive technologies and homecare services. Of these, homecare services were considered essential while volunteer support was the least preferred service. Most participants were keen on the usage of assistive technology but highlighted unwanted side effects.

### Perceptions of staff caregivers

One qualitative study [41] explored the perceptions of staff caregivers for PLWD. They concluded that although there has been an increase in understanding related to the role family caregivers play in caring for individuals with dementia, more research is needed to identify which strategies work best in which situations.

## Discussion

This scoping review provides a comprehensive overview of literature on caregiver support systems for PLWD and communication comorbidities. Although there was only one study [24] that included people living with hearing loss, it was a qualitative study that assessed the hearing loss burden and communication needs of participants and staff at a day health centre to create effective staff training and communication interventions. The burden and effects of hearing loss were studied to develop practical training programmes that enhanced communication. The findings can help improve communication in patients suffering from hearing impairment.

The relationship between interventions and communication emerged as a critical factor influencing caregiver burden. Interventions such as ADS, educational programs, and counselling were most effective when they addressed communication challenges explicitly. For example, ADS programs that included structured communication supports and staff training were associated with improved caregiver confidence and reduced stress [20, 28]. Similarly, educational interventions that taught communication strategies alongside behavioural management skills significantly reduced caregiver burden [32]. These findings highlight the need for integrated approaches that combine respite, education, and communication-focused strategies.

Adult day care services play a crucial role in alleviating caregiver burden for individuals caring for loved ones living with dementia. These services offer a safe and stimulating environment for individuals with dementia, providing them with structured activities and social interactions tailored to their cognitive abilities and interests. By entrusting their loved ones to adult day care facilities for a portion of the day, caregivers gain respite from their caregiving responsibilities, allowing them to attend to their own needs, such as work commitments, household tasks, or personal time for rest and rejuvenation. Studies have shown that utilising adult day care services can significantly reduce caregiver stress and depression while improving their overall well-being and quality of life [45]. Additionally, regular use of adult day care has been associated with delaying institutionalisation of individuals with dementia, enabling them to remain in their homes and communities for a longer duration [46]. Therefore, integrating adult day care services into dementia care plans can not only benefit the PLWD by providing them with engaging activities and socialisation but also support caregivers in managing the demands of caregiving more effectively.

Educational interventions are instrumental in reducing caregiver burden for individuals caring for loved ones with dementia. These interventions provide caregivers with essential knowledge and skills to effectively manage the challenges associated with dementia care, including communication strategies, behaviour management techniques, and self-care practices. By equipping caregivers with a better understanding of dementia symptoms and progression, as well as strategies for coping with caregiving stress, educational interventions empower caregivers to feel more confident and competent in their caregiving roles. Education can help caregivers anticipate and plan for future care needs, navigate healthcare systems more effectively, and access available support services. Research has demonstrated that participation in educational programs leads to improvements in caregiver knowledge, self-efficacy, and mental health outcomes, ultimately reducing caregiver burden [47]**.** Therefore, incorporating educational interventions into dementia care plans is essential for enhancing caregiver well-being and optimising the quality of care provided to PLWD.

Hearing and communication disability evaluation in PLWD can be beneficial in dementia care. Hearing interventions have been shown to have a beneficial effect on communication and cognition in dementia [48, 49]. A prospective cohort study found that cognitive performance was improved in hearing aid users, and hearing interventions could potentially delay onset of dementia [50]. Another study conducted in the USA noted that hearing interventions can delay cognitive decline in elderly patients [51]. These findings indicate that screening and early diagnosis and treatment of hearing impairments in PLWD can significantly improve cognitive outcomes and reduce communication disorders. However, the complexity of standard audiometry, which requires complex attention, learning and memory to complete the procedure has prevented its widespread use. The use of electrophysiological tests and behavioural approaches to diagnose hearing loss can be considered to overcome this limitation [52]. A dementia care plan that encompasses hearing and communication disability evaluation will also aid in reducing the burden on caregivers.

Speech-language pathologists (SLPs) play a pivotal role in dementia care by delivering both direct and indirect communication interventions. Direct approaches include structured language stimulation sessions such as group conversation therapy and cognitive stimulation activities, which are designed to strengthen word-finding ability and discourse skills in PLWD. Indirectly, SLPs train caregivers in tailored communication strategies (e.g. simplifying sentence structure, using multimodal cues, and pacing conversational turns) and introduce augmentative and alternative communication (AAC) aids to support comprehension and expression. Such interventions have been associated with significant improvements in caregiver—PLWD dialogue quality and reductions in caregiver burden [53].

Supportive technologies play a vital role in reducing caregiver burden. These technologies encompass a wide range of tools and devices designed to assist caregivers in various aspects of caregiving, including monitoring, safety, communication, and daily task management. For instance, GPS tracking devices can help caregivers keep track of individuals with dementia who may wander or become lost, providing peace of mind and enhancing safety. Remote monitoring systems allow caregivers to supervise their loved ones' activities and well-being from a distance, enabling timely intervention in case of emergencies or changes in health status. Communication aids, such as video calling apps and reminder systems, facilitate communication between caregivers and individuals with dementia, promoting social engagement and adherence to daily routines. Moreover, assistive technologies, such as electronic pill dispensers and automated home safety systems, help simplify medication management and reduce the risk of accidents in the home environment. By integrating supportive technologies into caregiving routines, caregivers can enhance their efficiency, alleviate stress, and improve the quality of care provided to individuals with dementia. Research has shown that the use of supportive technologies can lead to reduced caregiver burden and improved caregiver well-being, thereby supporting the sustainability of informal caregiving arrangements (REF) [54]. In terms of improving communication, information technologies, ranging from tablet-based AAC apps to social robots, have been shown to sustain engagement, and prompt exchanges in small studies, thereby reducing conversational pressure on caregivers and contributing to measurable declines in caregiver burden [55]. Therefore, incorporating these technologies into dementia care plans can complement traditional caregiving approaches and contribute to better outcomes for both caregivers and care recipients.

Caregiver counselling is a valuable resource in reducing caregiver burden, as it offers caregivers a supportive environment to express their feelings, concerns, and challenges related to caregiving, while also providing them with coping strategies and problem-solving skills.. Crucially, counselling programs equip caregivers with tailored communication techniques such as reframing questions, using reflective listening, and applying multimodal prompts to reduce misunderstandings and minimise frustration during care interactions, which in turn lowers perceived stress and overall burden. Additionally, counselling can help caregivers navigate complex family dynamics, address guilt or resentment, and adjust to the changing needs of those in their care. Research has demonstrated that caregiver counselling interventions lead to improvements in caregiver mental health outcomes, including reductions in depression, anxiety, and caregiver burden [56]. Therefore, integrating caregiver counselling into dementia care plans can enhance caregiver resilience, improve the quality of care provided to PLWD, and ultimately promote the well-being of both caregivers and care recipients.

While caregiver counselling is widely recognised as a valuable intervention for reducing caregiver burden in dementia care, it is important to critically assess its efficacy and limitations. Firstly, although counselling can offer emotional support and coping strategies to caregivers, its accessibility may be limited by factors such as cost, availability of qualified counsellors, and logistical barriers, especially for caregivers in rural or underserved areas. Additionally, the effectiveness of counselling interventions may vary depending on individual caregiver characteristics, such as their level of distress, coping style, and willingness to engage in counselling. Some caregivers may not perceive the need for counselling or may face stigma associated with seeking mental health support, which can hinder their willingness to participate in counselling sessions.

Furthermore, while research suggests that caregiver counselling can lead to improvements in caregiver mental health outcomes, including reductions in depression and anxiety, the magnitude of these effects may vary across studies, and long-term benefits may not always be sustained. Additionally, the evidence supporting the effectiveness of counselling in reducing caregiver burden specifically related to dementia care is mixed, with some studies reporting significant reductions in burden while others find more modest effects or no significant difference compared to control groups.

Moreover, caregiver counselling may not address broader systemic issues that contribute to caregiver burden, such as inadequate support services, financial strain, or limited access to respite care. To maximise the impact of counselling interventions, it is essential to integrate them within comprehensive dementia care programs that address the multifaceted needs of caregivers, including education, supportive services, and practical assistance. While caregiver counselling can be a valuable component of dementia care, its effectiveness may be influenced by various contextual factors, and it should be complemented by other support services to address the diverse needs of caregivers comprehensively. Future research should focus on identifying the most effective counselling approaches, tailoring interventions to individual caregiver needs, and integrating counselling within broader support frameworks to optimise caregiver outcomes in dementia care.

The adoption of multi-component caregiver support models such as the Resources for Enhancing Alzheimer’s Caregiver Health (REACH II) program can significantly reduce caregiver burden. REACH II delivers an individualized, six-month intervention combining skills training in problem-solving, stress and mood management, and both in-person and telephone coaching sessions. Importantly, it teaches caregivers to use structured communication strategies such as simplifying language, applying multimodal cues, and integrating augmentative and alternative communication aids, to facilitate clearer, more meaningful exchanges with PLWD [57]. Clinical evaluations of REACH II have documented significant improvements in caregiver–care-recipient dialogue quality and marked reductions in perceived caregiver burden.

### Limitations

This review has several limitations. First, communication profiles vary substantially by dementia subtype and disease stage, yet the included studies rarely stratified findings accordingly. This heterogeneity limits the generalisability of intervention effects. Second, restricting the review to English-language publications may have excluded relevant evidence, and publication bias remains a concern, as studies with positive outcomes are more likely to be published. Third, the diversity of intervention types, outcome measures, and reporting formats hindered direct comparisons and precluded subgroup analyses. Additionally, no formal quality appraisal was conducted, consistent with scoping review methodology [58], but this limits the ability to assess the robustness of individual studies. Finally, the review did not capture emerging digital interventions or informal community-based programmes not indexed in mainstream databases, and stakeholder input was not sought during scope definition or interpretation, which may affect the applicability of findings.

### Recommendations

It is recommended that a structured caregiver support system is developed and implemented to alleviate the considerable burden borne by caregivers of PLWD. As the specific needs of the caregiver and the person with dementia may vary, a combination of the strategies listed below should be considered in a comprehensive plan.

#### Educational support

These can improve understanding of dementia and communication comorbidities, as well as provide caregivers with the skills necessary to manage people under their care.

#### Communication skills training

These training programs can equip caregivers with communication strategies to enhance interaction with individuals with dementia and communication disorders.

#### Screening and evaluation services

Hearing and communication disorders must be screened and evaluated at an early stage to delay or prevent cognitive decline. This has an indirect effect on reducing caregiver burden.

#### Mental health support

Caregiver counselling and mental health support groups can offer resources for managing stress, anxiety, and depression. They can also act as platforms for caregivers to connect and learn from shared experiences.

#### Respite care services

These include adult day care centers, which can alleviate caregiver burden and provide temporary relief from caregiving duties.

In summary, there are various caregiver support systems for PLWD with communication comorbidities. While there is limited research exploring the specific challenges of hearing loss, other support systems have shown promise in improving caregiver well-being and care quality. Adult day care services could provide respite and social engagement for patients, and supportive technologies such as remote monitoring, tele-counselling, and companion robots could enhance safety, communication, and daily task management. Caregiver counselling offers emotional support and helps develop coping mechanisms. However, the accessibility, affordability, and effectiveness of these interventions vary and need to be studied further. Future research should explore developing and integrating such interventions within dementia care plans for a more comprehensive approach to caregiver well-being [59].

## Data Availability

No datasets were generated or analysed during the current study.

## Appendix

### Search strategy for Pubmed

Search date: Inception to February 28, 2024.

((("dementia"[MeSH Terms] OR "alzheimer disease"[MeSH Terms]) AND "communication disorders"[MeSH Terms]) OR "hearing loss, sensorineural"[MeSH Terms]) AND "caregivers"[MeSH Terms] AND "homes for the aged"[MeSH Terms]) OR "adult day care centers"[MeSH Terms].

Limits applied: English language only.

### Search strategy for embase

Search date: Inception to February 28, 2024.

(((((dementia or alzheimer disease) and communication disorders) or hearing loss) and caregivers and homes for the aged) or adult day care centers.mp.

Limits applied: English language only.

**Table:** Included studies grouped by geographical location

**Region**	**Country**	**Author (Year)**
North America	United States of America	Bonner (2014) [33]
		Marx (2016) [35]
		Tewary (2018) [34]
		Mamo (2018) [24]
		Logsdon (2016) [30]
		Boafo (2023) [28]
		Leggett (2016) [26]
		Ivey (2018) [23]
		Holden (2021) [42]
		Parker (2019) [22]
		Dabelko-Schoeny (2020) [41]
Europe	Spain	Tamayo-Morales (2021) [32]
		Maseda (2015) [31]
	Switzerland	Liu (2017) [25]
	Italy	DeCola (2016)
	United Kingdom	Orellana (2020) [27]
	Germany	vonKutzleben (2016)
		Behrndt (2019) [28]
	Norway	Rokstad (2018) [29]
		Faeo (2020) [40]
		Tretteteig (2016) [20]
Oceania	New Zealand	Liang (2017) [37]
Asia	Japan	Honjo (2020) [21]

**Table:** Included studies grouped by study design

**Study Design**	**Author (Year)**
Randomized controlled trial (RCT)/Pilot RCT/Quasi-experimental studies	Tamayo-Morales (2021) [32]
	Bonner (2014) [33]
	Tewary (2018) [34]
	Behrndt (2019) [38]
	Liang (2017) [37]
	Rokstad (2018) [29]
Cross-sectional study	Maseda (2015) [31]
	vonKutzleben (2016)
Cohort study	DeCola (2016)
	Logsdon (2016) [30]
	Liu (2017) [25]
	Ivey (2018) [23]
	Parker (2019) [22]
	Honjo (2020) [21]
	Leggett (2016) [26]
Qualitative study	Mamo (2018) [24]
	Marx (2016) [35]
	Boafo (2023) [28]
	Faeo (2020) [40]
	Holden (2021) [42]
	Dabelko-Schoeny (2020) [41]
Mixed methods research	Orellana (2020) [27]
Integrative review	Tretteteig (2016) [20]
